# The main and added effects of heat on mortality in 33 Chinese cities from 2007 to 2013

**DOI:** 10.1007/s11783-023-1681-5

**Published:** 2023-01-27

**Authors:** Yanlin Niu, Jun Yang, Qi Zhao, Yuan Gao, Tao Xue, Qian Yin, Peng Yin, Jinfeng Wang, Maigeng Zhou, Qiyong Liu

**Affiliations:** 1 Beijing Center for Disease Prevention and Control, Institute for Nutrition and Food Hygiene, Beijing 100013, China; 2 State Key Laboratory of Infectious Disease Prevention and Control, National Institute for Communicable Disease Control and Prevention, Chinese Center for Disease Control and Prevention, Beijing 102206, China; 3 University College London, London, WC1H 0NN, UK; 4 School of Public Health, Guangzhou Medical University, Guangzhou 511436, China; 5 Department of Epidemiology, School of Public Health, Cheeloo College of Medicine, Shandong University, Jinan 250012, China; 6 Shandong University Climate Change and Health Center, Shandong University, Jinan 250100, China; 7 Department of Epidemiology, IUF-Leibniz Research Institute for Environmental Medicine, Düsseldorf 40225, Germany; 8 School of Public Health and Preventive Medicine, Monash University, Melbourne, VIC 3004, Australia; 9 Institute of Reproductive and Child Health, Ministry of Health Key Laboratory of Reproductive Health and Department of Epidemiology and Biostatistics, School of Public Health, Peking University, Beijing 100191, China; 10 State Key Laboratory of Resources and Environmental Information System, Institute of Geographical Sciences and Nature Resources Research, Chinese Academy of Sciences, Beijing 100101, China; 11 National Center for Chronic and Noncommunicable Disease Control and Prevention, Chinese Center for Disease Control and Prevention, Beijing 100050, China; 12 University of Chinese Academy of Sciences, Beijing 100049, China

**Keywords:** Heat, Added effect, Vulnerable population, Main effect, Mortality

## Abstract

Increases in ambient temperatures and the frequency of extreme heat events constitute important burdens on global public health. However, evidence on their effects on public health is limited and inconclusive in China. In this study, data on daily deaths recorded in 33 Chinese cities from 2007 to 2013 was used to evaluate the effect of heat on mortality in China. In addition to the definition of a heatwave established by the China Meteorological Administration, we combined four city-specific relative thresholds (90th, 92.5th, 95th, and 97.5th percentiles) of the daily mean temperature during the study period and three durations of ⩾ 2, ⩾ 3, and ⩾ 4 days, from which 13 heatwave definitions were developed. Then, we estimated the main and added effects of heat at the city level using a quasi-Poisson generalized additive model combined with a distributed lag nonlinear model. Next, the estimates for the effects were pooled at the national level using a multivariable meta-analysis. Subgroup analysis was performed according to sex, age, educational attainment, and spatially stratified heterogeneity. The results showed that the mortality risk increased from 22.3% to 37.1% due to the effects of the different heatwave definitions. The added effects were much lower, with the 2 Front. Environ. Sci. Eng. 2023, 17(7): 81 highest increase of 3.9% (95% *CI*: 1.7%–6.1%) in mortality risk. Females, the elderly, populations with low educational levels, and populations living inland in China were found to be the most vulnerable to the detrimental effects of heat. These findings have important implications for the improvement of early warning systems for heatwaves.

## Graphical abstract

**Graphic abstract:**
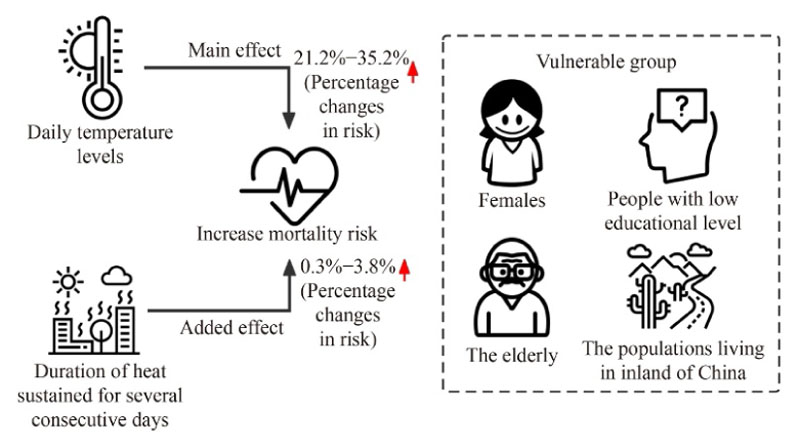

## 1 Introduction

Extreme heat events with longer durations, higher frequencies, and greater intensities have become increasingly common due to climate change (Hoegh-Guldberg et al., 2018; Lange et al., 2020; Zhao et al., 2021). At present, the number of individuals exposed to extreme heat is projected to substantially increase with each additional unit of warming (Byers et al., 2018; Romanello et al., 2021), with a growing number of deaths due to heat exposure expected on a global scale. A clear increasing trend in heatwave-related mortality has been observed in China during the past two decades, with an increase of 63.6% in 2015–2020 compared to 2000–2004 (Cai et al., 2021). Although humanity’s ability to adapt to extreme temperatures is likely to improve in the future, heat-related excess mortality continues to rise with rising temperatures, with projections indicating an increase from 1.9% in 2010 to 2.4% in 2030 and 5.5% in the 2090 in China (Sun et al., 2021; Yang et al., 2021). Thus, the negative health effects of exposure to extreme heat have become a non-negligible threat to human beings. In this context, further research on the health impact of heat is needed, especially in developing countries, to provide evidence for the development of mitigation and adaptation strategies (Campbell et al., 2018; Vicedo-Cabrera et al., 2021).

The impact of heat on human health is commonly divided into two categories (Hajat et al., 2006; Gasparrini and Armstrong, 2011): the main effect, which is caused by daily temperature levels and estimated by the usual exposure-response relationship between temperature and health on both heatwave days and non-heatwave days, and the added effect due to the duration of heat sustained for several consecutive days, which is estimated by the time of duration and specific temperatures (i.e., extreme temperatures during a heatwave). The majority of studies in this field have found that the main and added effects of heat significantly increase the health risk of exposure to extreme heat, wherein the impact of latter is relatively small compared to the former (Gasparrini and Armstrong, 2011; Lee et al., 2016; Sherbakov et al., 2018; Yin et al., 2018). However, some researchers have failed to identify the added effects in their studies (Barnett et al., 2012; Arbuthnott and Hajat, 2017; Guo et al., 2017). Possible reasons for these inconsistent conclusions are as follows: (1) different study areas and populations resulted in various influencing factors of vulnerability, including climatic characteristics, local socioeconomic level, and capacity of residents to adapt to changes in climate (Hajat and Kosatky, 2010; Yin et al., 2018; Sera et al., 2019; Yang et al., 2019; Ebi et al., 2021); (2) diverse study designs, including different temperature metrics, heatwave definitions, health outcomes, and models and parameters (Chen et al., 2015; Arbuthnott and Hajat, 2017; Yang et al., 2019); (3) heat acclimatization may change with time and the implementation of adaptation strategies (Jay et al., 2021). This highlights the fact that current research in this area is insufficient to satisfy the needs of policymakers, especially in certain regions (e.g., South Asia and China) (Dimitrova et al., 2021).

Furthermore, evaluations at the individual level and in different areas is also of great importance for formulating targeted strategies and tailored measures to respond to global warming (Anderson and Bell, 2011; Son et al., 2019). Therefore, gathering evidence is crucial to fill this gap and develop credible solutions for reducing the exposure of vulnerable populations to the health risks of extreme.

In this study, a database of temperature, mortality, and air pollution in 33 Chinese cities from 2007 to 2013 was established. The primary objective of this study was to quantify and compare the main and added effects of heat under multiple heatwave definitions. Second, considering the large influence of individual-level characteristics, including sex, age, and education, on the relationship between heat and health (Son et al., 2019), we aimed to further evaluate the effect modification of these factors. Finally, the spatially stratified heterogeneity of effects in different areas was examined.

## 2 Materials and methods

### 2.1 Study sites

The study population included residents from 33 major cities in China, including 31 provincial capital cities that have been described in a previous study (Yang et al., 2019), as well as Shenzhen and Ningbo. The study sites covered all the different climatic and geographical zones in China, which can be used as representative samples. According to the Chinese North–south demarcation (Tan, 2011) and coastal administrative area classification and codes (Center, 2006), the study sites were divided into south and north and categorized into coastal cities and inland cities (Table 1). The study period was from 2007 to 2013.

### 2.2 Data collection

Data on the daily number of deaths of urban residents from 2007 to 2013 were obtained from the Chinese National Center for Chronic and Noncommunicable Disease Control and Prevention. Based on the 10th revision of the International Classification of Diseases (ICD-10), records were included if the underlying cause of death was non-accidental (ICD-10: A00-R99). The daily number of deaths was further categorized by sex, age group (0–64, 65–74 and ⩾ 75 years) (Yin et al., 2018; Yang et al., 2019), and educational attainment (illiterate, primary school, and junior high school and above). The annual population for each city was obtained from the National Bureau of Statistics.

Daily contemporaneous meteorological data were obtained from the ERA5 data set, published by ECMWF (European Centre for Medium-Range Weather Forecasts), including the daily mean temperature (°C), daily maximum temperature (°C), daily minimum temperature (°C), relative humidity (%), and average wind velocity (m/s). ERA5 reanalysis is a climate data set containing many atmospheric, land surface, and sea state parameters produced by the ECMWF with in situ and satellite observations (Hersbach et al., 2020). ERA5 data cover the period from 1950 to the present and are available on regular latitude-longitude grids at approximately 31 km × 31 km resolution (0.25° × 0.25°). The daily series of meteorological indicators for each city in this study were obtained by calculating the spatial average of values in grid points weighted by the proportion of the area of the city covered by the grid.

The daily mean concentrations of PM_2.5_ at the city level were obtained from the PM_2.5_ Hindcast Database (PHD). The PHD is a database that assembles data sets from multiple sources using a machine learning approach, which provides historical PM_2.5_ estimates in a regular grid of 0.1° × 0.1° across China, from 2000 to 2016 (Xue et al., 2019). The data processing of PM_2.5_, which was the same as the meteorological data, and the mean value within the study area were taken as the average exposure level of air pollution.

### 2.3 Heatwave definition

At present, there is no unified definition for “heatwave” (Chen et al., 2015; Tong et al., 2015; Xu et al., 2016; Guo et al., 2017; Yang et al., 2019). In addition, it is inappropriate to use a single definition because of the diverse adaptability of individuals from different regions to changes in their environment and climate (Yang et al., 2019). In China, heatwaves are defined by the China Meteorological Administration (CMA) as three or more consecutive days with daily maximum temperatures exceeding a threshold of 35 °C. In addition to the definition proposed by the CMA, we further established four relative thresholds (90th, 92.5th, 95th, and 97.5th percentiles) based on the statistical distributions of daily mean temperature during the study period (Guo et al., 2017) and three durations of ⩾ 2, ⩾ 3, and ⩾ 4 days. As a result, a total of 13 definitions for “heatwave” were established (Table 1).

### 2.4 Statistical analysis

#### 2.4.1 Two-stage analysis strategy

In this study, we used a two-stage analysis strategy to assess the effects of heat on mortality. In the first stage, city-specific associations between heat and mortality, including the main and added effects, were estimated. In the second stage, the estimates for associations among the cities studied were pooled at the national level using multivariable meta-analysis. The analyses were restricted to the warm season (from May to September), which was considered to be the prime period of high temperatures and heatwaves in China in previous studies (Chen et al., 2015; Yin et al., 2018; Yang et al., 2019). The analysis was conducted separately for each heatwave definition.

In the first stage, we used a quasi-Poisson generalized linear model that assumed the mortality count to follow an over-dispersed distribution for each day to estimate the city-specific mortality risk (relative risk, RR) from heat by including temperature and a heatwave indicator (Gasparrini and Armstrong, 2011), as follows:

$$\begin{array}{l} \log[E(Y_{t})]=\alpha +ns(RH_{t},3)+ns(year_{t},5)+ns(doy_{t},4)\\ +\gamma *DOW_{t}+\delta *HOLIDAY_{t}+offset(\log(pop))\\ +\beta *Temp_{t,l}+\varepsilon *HW_{t} \end{array}$$

where *E*(*Y*_*t*_) is the expected number of deaths on day *t, α* is the intercept, and ns(·) is the natural cubic spline function. Relative humidity on day *t* (*RH*_*t*_) with three degrees of freedom (df) was used to control for potential confounding effects (Hu et al., 2020). Based on previous studies (Gasparrini and Armstrong, 2011; Lee et al., 2018), year (*year*_*t*_) with 5 df and day of the year (*doy*_*t*_) with 4 df were used to control for long-term and seasonal trends, respectively. The days of the week (*DOW*_t_) and public holidays (*HOLIDAY*_*t*_) were also included in the model as categorical variables, with the corresponding coefficients of *γ* and *δ*. Each year’s population for each city on a log scale was included in the model as an offset to control for the potential confounding effect of demographic shifts over time (Qiao et al., 2015; Tong et al., 2015; Cheng et al., 2018). *Temp*_*t*,*l*_ is the cross-basis matrix of the daily mean temperature on day *t* to estimate the nonlinear effect and lag effect, and *l* is the lag days. *β* is the vector of the regression coefficients for *Temp*_*t*,*l*_. The relationship in the temperature space was modeled by a natural cubic spline with 6 df and three equally spaced knots, while changes in the shape along lags were modeled by a natural cubic spline with 5 df, up to a maximum lag day of 10 (Gasparrini and Armstrong, 2011). *HW*_*t*_, with the corresponding coefficient of *ε*, was the dummy variable, assigned 1 for days with heatwaves and 0 for days without heatwaves, based on the heatwave definitions mentioned in Section 2.3. The temperature with the minimum mortality risk for each city was used as the reference value for estimating the RR of temperature, which was derived from the best linear unbiased prediction (BLUP) of the overall cumulative exposure-response association between daily mean temperature and mortality (Gasparrini et al., 2015). Based on previous studies (Hajat et al., 2006; Guo et al., 2017), the main effect was defined as the independent impact of daily temperature on health, whereas the added effect was defined as an additional risk due to the duration of heat sustained for several consecutive days. Then, the city-specific main effect was estimated as the RR between the median temperature among heatwave days and the minimum mortality temperature using *Temp*_*t*,*l*_, and the city-specific added effect was estimated as the exponential of the coefficient for *HW*_*t*_ (Gasparrini and Armstrong, 2011). The main and added effects are presented as percentage changes in the risk.

In the second stage, the pooled estimates of the main and added effects were produced using a multivariable meta-analysis based on restricted maximum likelihood (REML). Heterogeneity between cities was evaluated by calculating I-square (*I*^2^) statistics and p-values using the Cochran Q test in meta-regression models (Gasparrini et al., 2012). *I*^2^ represents the percentage of variability in the RRs attributable to the cities.

The Akaike information criterion for quasi-Poisson (Q-AIC) was used to assess the goodness of the model fit among the 13 heatwave definitions. The sum of the Q-AIC values for each heatwave definition from all models in the 33 cities was compared. Then, the optimal model fit and optimal heatwave definition were determined when the sum was minimized.

#### 2.4.2 Subgroup analysis

To evaluate the effect modification of factors at the individual level and to identify vulnerable subpopulations, the aforementioned two-stage analysis was repeated by sex, age group, and educational attainment (Yin et al., 2018; Yang et al., 2019). The difference between the effect estimates for the two subpopulations was compared, and the significance was tested using the following formula:

$$Z=\frac{E_{1}-E_{2}}{\sqrt{SE{\left(E_{1}\right)}^{2}+SE{\left(E_{2}\right)}^{2}}}$$

where *Z* is the *Z*-test value, *E*_1_ and *E*_2_ are the effect estimates for the two subgroups, and *SE*(*E*_1_) and *SE*(*E*_2_) are their respective standard errors (Clogg et al., 1995; Paternoster et al., 1998).

#### 2.4.3 Spatial stratified heterogeneity analysis

Spatial stratified heterogeneity is a major feature of geographical objects, which refers to a within-strata variance that is less than the between-strata variance. Geodetector is widely used to explore and utilize spatial heterogeneity, and its core idea is based on the assumption that there would be similarity in the spatial distribution of certain independent variables and their corresponding dependent variables if the former had a significant impact on the latter (Wang et al., 2010; Wang and Hu, 2012). The factor detector in Geodetector can explore the spatial heterogeneity of variable *Y* and quantify the extent to which factor *X* explains the heterogeneity of attribute *Y* (Wang et al., 2010). The q-statistic method was proposed by Wang et al. (2010; 2016) to measure the degree of spatially stratified heterogeneity and to test its significance using the following expression:

$$\begin{array}{c} q=1-\frac{\sum_{h=1}^{L}N_{h}{ó}_{h}^{2}}{N{ó}^{2}}=1-\frac{SSW}{SST}\\ SSW=\sum_{h=1}^{L}N_{h}{\text{ó}}_{h}^{2}\\ SST=N{\text{ó}}^{2} \end{array}$$

where *h* = 1, 2, …, *L* is the stratum of variable *Y* or factor *X* in the area, the area is composed of *N* units, stratum *h* is composed of *N*_*h*_ units, and *σ*__h__^2^ and *σ*^2^ denote the variance of *Y* in stratum *h* and the entire area, respectively. *SSW* and *SST* represent the within-sum of squares and the total sum of squares, respectively. The range of the *q* value is 0 to 1, where the larger the value, the more obvious the spatial heterogeneity of *Y* and the stronger the explanatory power of factor *X* to attribute *Y* if the stratum is determined by factor *X*.

In this study, taking the mortality risk estimates (*β* for the main effect and *ε* for the added effect) as *Y* and the divisions mentioned in Section 2.1 as *X*, the spatial heterogeneity of the health impacts from heat was explored using Geodetector. In addition, the effects within the stratum were pooled using meta-analysis when heterogeneity was detected and compared. To decrease the probability of producing a false negative, two-tailed *P*-values less than 0.1 was considered statistically significant in this part of the analysis.

#### 2.4.4 Ethical approval

Ethical approval for this study was obtained from the Chinese Center for Disease Control and Prevention Ethical Review Committee (ICDC-2019008) prior to data collection. All analyzed data were anonymized and protected by a confidentiality agreement. This study was performed in accordance with the principles of the Declaration of Helsinki.

#### 2.4.5 Sensitivity analysis

To evaluate the robustness of the model, a sensitivity analysis was performed by modifying the df of the variables for the city-specific model, including the relative humidity (*df* = 3–5), year (*df* = 3–5), and day of year (*df* = 3–5). The maximum lag days for *Temp*__*t*,*l*__ were set to 7 and 14. Moreover, the variable PM__2.5__ was added to the model to test its influence on the heat effect. Sensitivity analyses were conducted in models with the mildest (*HW*1) and strictest (*HW*12) heatwave definitions

All statistical analyses and plots were conducted using the “dlnm” (Gasparrini, 2011), “metafor” (Viechtbauer, 2010), and “ggplot2” (Wickham, 2016) packages in R (version 3.6.3). Spatial heterogeneity analyses were implemented using Geodetector. For all statistical tests except the spatial heterogeneity analysis in Section 2.4.3, a two-tailed *P*-value less than 0.05 was considered as statistically significant.

## 3 Results

### 3.1 Descriptive statistics

The medians of the daily mean temperature, relative humidity, and PM__2.5__ concentration in the study area during the warm season from 2007 to 2013 were 23.39 °C, 74.45%, and 46.62 μg/m^3^, respectively (Table 2). During the study period, the total number of deaths was 2,097,942. The average number of deaths per day ranged from 2 in Lhasa to 239 in Chengdu (Table 2). The average number of daily deaths in males was 35, which was higher than that in females. Among all age groups, the average number of daily deaths reached a maximum of 29 in individuals aged 75 years or above. The average number of daily deaths in populations with an educational attainment of illiterate, primary school, and secondary school or higher was 13, 19, and 22, respectively (Table 2).

The number of heatwave days varied with different heatwave definitions (Table 1). The total heatwave days decreased with the increase in temperature threshold in the heatwave definition (e.g., *HW*1, *HW*4, *HW*7, and *HW*10) and showed a similar declining trend with the extension of duration under the same threshold (e.g., *HW*1, *HW*2, and *HW*3). During the study period, the maximum total number of heatwave days was 2,112 days under the loosest definition (i.e., *HW*1, in which the threshold of the daily mean temperature was ⩾ 90th percentile and the duration was ⩾ 2 days). Conversely, the most stringent definition (i.e., *HW*12, in which the threshold of the daily mean temperature was ⩾ 97.5th percentile and the duration was ⩾ 4 days) resulted in a minimum of 137 heatwave days. According to the definition proposed by the CMA, the total number of heatwave days was 381. The sum of Q-AIC for each heatwave definition from all models in 33 cities was a minimum of 229,422.7 under the definition of *HW*8, in which the threshold of the daily mean temperature was ⩾ 95th percentile and the duration was ⩾ 3 days (Table 1).

### 3.2 Effects estimation

**Fig. 1:**
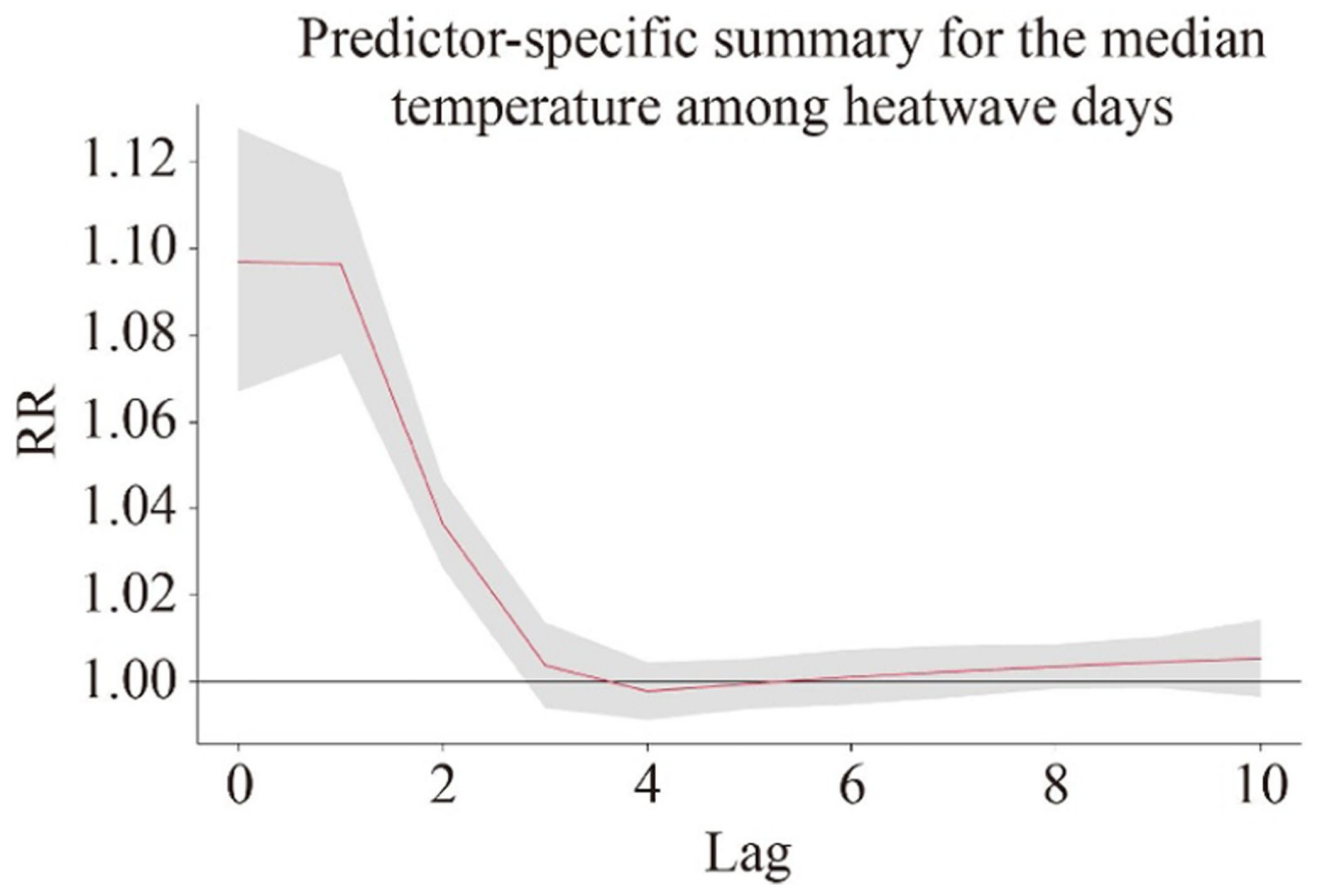
Lag pattern for the median temperature among heatwave days (28.42 °C) versus the minimum mortality temperature (21.10 °C) when adopted *HW*8.

The main effects of heat on mortality showed an upward trend with an increase in the temperature threshold and/or the extension of duration in the heatwave definition, varying from 22.3% to 37.1% with statistical significance (Table 3). The mortality risk increased by 28.3% (95% *CI*: 22.1%–34.9%) because of the main effect under the heatwave definition of *HW*8. In contrast to the main effect, the added effect decreased slightly with an increase in duration in the heatwave definition when the temperature threshold was relatively high, but increased with the increase in temperature threshold. Compared with the main effects, the added effects were much lower, with the highest increase of 3.9% (95% *CI*: 1.7%–6.1%) in mortality risk under the heatwave definition of *HW*10. When *HW*8 was adopted, the main effect was statistically significant from the exposure day to the 2nd day, reaching the highest increase of 9.7% (95% *CI*: 6.7%–12.8%) on the exposure day and then dropping dramatically from lag day 1 (Fig. 1).

### 3.3 Subgroup analysis

**Fig. 2:**
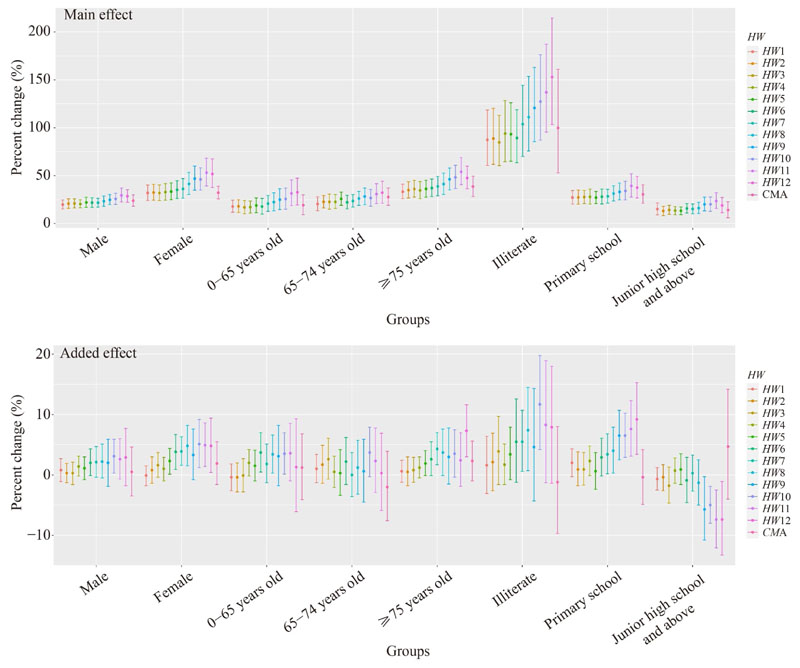
Subgroup comparison of the effect of heat on mortality risk under different heatwave definitions in 33 major cities in China from 2007 to 2013.

The effects of heat on mortality risk in the subgroups differed with the change in heatwave definition (Fig. 2, Table 3). Briefly, the main effect in subgroups increased with the increase in threshold temperature and/or the extension of duration in the heatwave definition. When the optimal heatwave definition *HW*8 was adopted in the model, the main effect increased the mortality risk in females by 41.4% (95% *CI*: 30.4%–53.4%), which was significantly higher than that in males (23.3%, 95% *CI*: 18.0%–28.8%; *Z* = *−*2.92, *P* = 0.004) (Table 4); the mortality risk increased by 41.1% (95% *CI*: 30.5%–52.5%) in individuals aged 75 years old or above, which was the highest in age groups (*Z* = *−*2.28, *P* = 0.023; *Z* = *−*2.55, *P* = 0.011); the mortality risk decreased when the education level improved, and illiterate individuals were more affected compared to individuals with superior educational attainments (*Z* = 4.82, *P* < 0.001; *Z* = 6.16, *P* < 0.001), which represents an increase of 111.1% (95% *CI*: 75.7%–153.5%) for risk. The variation in the added effect was not as regular as that in the main effect. Although the added effect showed a similar trend to the main effect on mortality risk in subgroups when adopting *HW*8, statistical significance was only observed when comparing the illiterate groups and the population with secondary or higher educational attainment (*Z* = 2.23, *P* = 0.026) (Table 4).

### 3.4 Spatial stratified heterogeneity analysis

No statistical significance was observed for the spatial stratified heterogeneity (SSH) of the heat effect between the north and south (Tables 4 and 5). Similarly, the SSH of the main effect between coastal and inland cities was not detected (Table 4). Regarding the added effect, the SSH was statistically significant between coastal and inland cities under the definition of *HW*10 (*q* = 0.1469, *P* = 0.0606) (Table 4). When pooling the effect within the stratum, the mortality risk in coastal cities increased by up to 1.1% (95% *CI*: −2.9%−5.2%) due to the added effect. The risk in inland cities increased by 4.9% (95% *CI*: 2.4%–7.5%). The increase in mortality risk in inland cities was much higher than that in coastal cities. However, no statistically significant added effect was observed in coastal cities (Table 5).

### 3.5 Sensitivity analysis

The results of the sensitivity analysis for both the main and added effects showed that the model was robust when the *df*s were altered for relative humidity (*df* = 3–5), year (*df* = 3–5), and day of year (*df* = 3–5) in the model (Fig. S1, Table S6). Changing the maximum lag days to 7 and 14 in the model did not result in significant differences in the fitting effect of the model (Fig. S2, Table S6). Furthermore, the exposure-response relationship was similar before and after adjusting for PM_2.5_ (Fig. S3, Table S6).

## 4 Discussion

In this study, we found that both the main effect from the increase in temperature and the added effect from sustained heat over several consecutive days increased the mortality risk, of which the former was much larger than the latter. Among the populations studied, females, the elderly, populations with low educational levels, and populations living inland in China were found to be more vulnerable to heat.

Consistent with previous studies (Campbell et al., 2018), our study found that high temperature could increase mortality risk significantly, which was defined as the main effect in this study. Similar to other studies (Guo et al., 2011; Guo et al., 2014), this effect appeared quickly and did not last for long. Moreover, an increasing number of studies have focused on the effects of heatwaves. Studies conducted in the US (Hajat et al., 2006; Gronlund et al., 2014; Sherbakov et al., 2018), China (Dong et al., 2016; Yin et al., 2018), Korea (Lee et al., 2016), and Iran (Aboubakri et al., 2019) have reported that the added effects could increase the risk of hospital admission and/or mortality from various diseases to varying degrees. Our results suggest that the main effects of heat could significantly increase the mortality risk in China, varying from 22.3% to 37.1%, whereas the added effects would only increase the mortality risk by up to 3.9%. Gasparrini and Armstrong (2011) found that the main effects of heat on mortality risk in the US were 4.9%–8.0%, while the added effects were 0.2%–2.8%, which was consistent with the results of the present study, wherein the added effect was found to be relatively smaller than the main effect. Using the definition of a heatwave in which the threshold of the daily mean temperature was ⩾ 97.5th percentile and the duration was ⩾ 3 days, Yin et al. (2018) found that the mortality risk in China increased by 7% due to the added effect, which is slightly higher than that observed in the present study. It is worth noting that the limited heatwave days identified in a relatively short period of three years in the study by Yin et al. may have resulted in the differences observed in conclusions between studies. Another study (Lee et al., 2016) in Seoul found that the added effect was 3.7%–18.1%, which was higher than observed that in the present study. This may be caused by the differences between Korea and China in terms of living habits and the climate adaptation ability of the residents and the natural environment. However, a multicountry study (Guo et al., 2017) failed to detect the added effect of heat in China. This may be due to the fact that it only included six cities in China. In contrast to previous studies (Dong et al., 2016; Guo et al., 2017; Yin et al., 2018) conducted in China, in the present study, a large dataset covering 33 cities over a long period of seven years was used, which provides a stronger basis for the provision of credible evidence to fill the gaps in this research area than previous studies.

In this study, females and the elderly, especially those with less education, were found to be more vulnerable to heat, consistent with the results of previous studies (Gronlund et al., 2014; Chen et al., 2015; Dong et al., 2016; Yin et al., 2018). Because of the difference in physiological and thermoregulation ability between sexes, females have a lower tolerance and adaptation to heat, which makes them more vulnerable (Druyan et al., 2012; Kim and Kim, 2017). A decline in body functioning and a high prevalence of chronic diseases in the elderly make it more likely and easier to induce or aggravate certain diseases during heatwaves, and even death (Gasparrini and Armstrong, 2011; Huang et al., 2022). People with less education usually have poor social and economic conditions, lack adequate and effective protective measures, and lack sufficient awareness of self-protection against adverse weather events, resulting in higher health impacts from heat exposure. The identification of vulnerable populations can help in formulating targeted public health interventions and strengthen health protection for key groups when meteorological disasters, such as heatwaves, occur. In this study, we also found that there was a strong spatially stratified heterogeneity of the health impacts from heat between coastal and inland areas in China, and the added effect was higher in inland areas than in coastal areas. A possible reason for this spatial heterogeneity may be the combined effect of natural and socio-economic factors in different regions. Compared with inland areas, coastal areas have better climatic conditions and ecological environments thanks to the influence of the ocean, which may alleviate some of the effects caused by high temperatures and heatwaves. In addition, benefiting from the more developed economies of coastal areas, local residents have better living conditions and abundant public resources, which enables them to adapt more easily to extreme weather events, such as heatwaves. However, we did not observe any spatial heterogeneity in terms of heat effects between the north and south of China.

Furthermore, we also found that the health effects of exposure to extreme heat varied with the different heatwave definitions, highlighting the need to exercise caution when choosing the threshold for defining heatwaves in early warning systems. To be specific, a looser definition for heatwaves would trigger the warning system much more easily, which can be more effective in reducing the adverse impacts of heatwaves on human health, but it may also result in a waste of public resources and public indifference, and vice versa. Moreover, a stricter definition that reflects the most intense heatwave, but also leads to the shortest heatwave period, may not necessarily have the strongest effect on reducing health risks (Heo et al., 2019). Therefore, it is of great importance to identify an optimal heatwave definition for use in early warning systems based on scientific evidence and local conditions, as reported in previous studies (Yin et al., 2018; Yang et al., 2019). In this study, the best model fit was observed when adopting the heatwave definition in which the threshold of the daily mean temperature was ⩾ 95th percentile and the duration was ⩾ 3 days, which indicated that this definition could best capture the health impact from heat. In contrast, a poor model fit was observed when the definition established by the CMA was adopted. In contrast to the results of this study, Yang et al. (2019) used the daily maximum temperature as the metric to determine the optimal heatwave definition in 31 cities in China, and obtained the best results when the threshold of the daily maximum temperature was ⩾ 92.5th percentile and the duration was ⩾ 3 days. Similarly, both the World Meteorological Organization (WMO) and CMA use the daily maximum temperature to define the heatwave. However, to better capture the impact of heatwaves on health, we conducted a trial test using four temperature metrics (daily mean temperature, daily maximum temperature, daily minimum temperature, and apparent temperature) in the exposure-response function with the same parameters introduced in the two-stage analysis strategy in Section 2.4.1, and compared the model fits using Q-AIC, which is presented in detail in Table S7. The results of this trial test showed that daily mean temperature was the optimal metric. It is worth noting that the temperature metrics used in heatwave-related studies vary across the literature (Gasparrini and Armstrong, 2011; Chen et al., 2015; Dong et al., 2016; Lee et al., 2016; Yin et al., 2018; Heo et al., 2019; Yang et al., 2019). Guo et al. (2017) found that the exposure-response relationships between heatwaves and mortality were similar when using the daily mean temperature and the daily maximum temperature in their definition of a heatwave, but better than that when using the daily minimum temperature. Moreover, several thermal comfort indices, which quantify the combined effect of a series of meteorological factors (e.g., air temperature, humidity, and wind speed) on perceived temperature, were compared for health risks using the heatwave definition (Heo et al., 2019). They found that web-bulb globe temperature (WBGT) was better associated with a significant risk of mortality than air temperature and heat index (HI). Furthermore, Nori-Sarma et al. highlighted the issue of whether to use an absolute metric or a relative metric as the temperature metric when assessing the relationship between heat and health (Nori-Sarma et al., 2019). However, the choice of heatwave definition depends on the tradeoffs of many factors, and will need to be updated as humans change their ability to adapt to extreme heat (Heo et al., 2019; Yang et al., 2019). In this study, except for the heatwave definitions used by the CMA, four temperature metrics, four temperature thresholds, and three durations of heatwaves were combined to develop different heatwave definitions, providing insights into the significance of the definition of heatwaves when assessing the health effects of heat.

In this study, the main effect showed a rising trend with an increase in the threshold temperature and a prolonged duration, while the added effect increased with the increase in threshold temperature but decreased slightly with the extension of duration when the temperature threshold was relatively high. Similar to our findings, Yin et al. (2018) found that the added effect of heatwaves in China increased with an increase in the temperature threshold, but a relationship between the added effect and the duration of heatwaves was not observed. Another study (Guo et al., 2017) also supported this conclusion. However, some studies (Díaz et al., 2002; Anderson and Bell, 2011; Son et al., 2012; Dong et al., 2016) have indicated that the temperature threshold and duration in the heatwave definition could modify the health impact of heat. These differences among studies may be caused by the lack of full consideration of the lag effect of heatwaves in the models (Yin et al., 2018), which should be given more attention in the future.

This study had some limitations. First, the data on daily deaths were limited to cities, but influencing factors, such as the economic development level, medical condition, and protective awareness of the residents, may lead to differences in the health effects of heat between urban and rural areas (Hu et al., 2019). Therefore, whether the conclusions of this study are suitable for rural regions will need to be studied further. Second, the meteorological data used in this study were obtained from the reanalysis dataset, which does not represent the actual exposure level of individuals, such that exposure measurement error is inevitable. Finally, the study failed to control for influencing factors at the individual level, such as the use of air conditioning and hygiene level, which may have caused biases in the results.

## 5 Conclusions

The main and added effects of heat co-exist in China, both of which could increase the mortality risk in populations exposed to extreme heat events, wherein the main effect had a greater impact. Females, the elderly, populations with low educational levels, and individuals living inland in China were the most vulnerable to extreme heat. These findings highlight the need for public health departments to accelerate the establishment and improvement of early warning systems for heatwaves and strengthen health education and health promotion, especially among the vulnerable groups identified in this study.

## Supplementary Material

**Electronic Supplementary Material** Supplementary material is available in the online version of this article at https://doi.org/10.1007/s11783-023-1681-5 and is accessible for authorized users.

Supplementary Material

## Figures and Tables

**Table 1:** Summary of heatwave definitions and heatwave days in 33 main cities in China in warm season from 2017 to 2013

Heatwave definition	Threshold	Duration	Total	Mean	Standard deviation	Min.	Median	Max.	Q-AIC
HW1	⩾ 90th	⩾ 2d	2112	64	8	50	61	82	229431.3
HW2	⩾ 90th	⩾ 3d	1313	40	10	24	36	62	229480.3
HW3	⩾ 90th	⩾ 4d	864	26	10	10	23	50	229382.8
HW4	⩾ 92.5th	⩾ 2d	1494	45	7	31	42	59	229433.0
HW5	⩾ 92.5th	⩾ 3d	886	27	8	13	24	44	229452.1
HW6	⩾ 92.5th	⩾ 4d	553	17	7	5	14	33	229448.5
HW7	⩾ 95th	⩾ 2d	915	28	5	18	26	40	229355.9
HW8	⩾ 95th	⩾ 3d	517	16	6	6	14	29	229422.7
HW9	⩾ 95th	⩾ 4d	302	9	5	1	8	22	229439.3
HW10	⩾ 97.5th	⩾ 2d	428	13	3	7	12	19	229417.1
HW11	⩾ 97.5th	⩾ 3d	239	7	4	2	7	14	229371.7
HW12	⩾ 97.5th	⩾ 4d	137	4	3	0	4	11	229427.5
CMA	35°C	⩾ 3d	381	12	20	0	0	76	229487.6

Note: The city-specific relative thresholds based on the statistical distribution of daily mean temperature during the study period were used from HW1 to HW12, while the absolute threshold of daily maximum temperature was used by CMA.

**Table 2:** Statistical description of mortality and environmental indicators in 33 main cities in China in warm season from 2007 to 2013

Items	Total	Min.	P25	Median	Mean	P75	Max.
Mortality (Count)	2097942	0	13	30	60	88	805
Sex							
Male	1229326	0	8	19	35	51	445
Female	868616	0	5	12	25	37	360
Age (Years)							
< 65	594975	0	4	10	17	24	184
65−74	403276	0	2	6	12	17	164
⩾ 75	1003259	0	5	14	29	43	429
Educational attainments							
Illiterate	471080	0	1	5	13	17	309
Primary school	661048	0	3	9	19	27	319
Secondary or higher	762046	0	5	12	22	29	152
Daily mean temperature (°C)		−2.84	19.16	23.39	22.36	26.85	35.20
Relative humidity (%)		14.42	62.72	74.45	70.73	81.79	98.27
PM_2.5_ (μg/m^3^)		1.47	31.61	42.62	47.39	58.46	196.45

Note: P25 and P75 refer to 25th and 75th percentiles, respectively.

**Table 3:** Pooled main and added effects for mortality risk with tests for heterogeneity across cities with different heatwave definitions

Heatwave definition	No. Cities	Main effect				Added effect		
		Percentage change (%) (95% *CI*)	*I* ^2^	*P*		Percentage change (%) (95% *CI*)	*I* ^2^	*P*
*HW*1	33	22.3 (17.8, 27.0)	46.77	< 0.001		0.2 (−1.4, 1.9)	34.59	0.017
*HW* 2	33	23.7 (18.4, 29.2)	55.10	< 0.001		0.4 (−1.1, 1.9)	12.89	0.185
*HW* 3	33	24.0 (18.8, 29.4)	52.86	< 0.001		0.6 (−1.2, 2.5)	23.68	0.017
*HW* 4	33	22.6 (17.6, 27.8)	50.40	< 0.001		1.2 (−0.1, 2.6)	< 0.01	0.819
*HW* 5	33	24.4 (19.0, 30.0)	52.83	< 0.001		1.5 (0, 3.1)	0.02	0.203
*HW* 6	33	24.6 (19.4, 30.2)	51.49	< 0.001		2.3 (0, 4.7)	28.04	0.016
*HW* 7	33	25.8 (20.4, 31.5)	46.18	< 0.001		2.7 (0.9, 4.5)	15.24	0.272
*HW* 8	33	28.3 (22.1, 34.9)	54.48	< 0.001		2.6 (0, 5.4)	39.61	0.010
*HW* 9	33	31.3 (25.2, 37.7)	44.17	< 0.001		1.0 (−2.8, 4.9)	53.16	< 0.001
*HW* 10	33	32.3 (25.8, 39.3)	44.88	< 0.001		3.9 (1.7, 6.1)	4.40	0.177
*HW* 11	33	37.1 (29.3, 45.3)	52.23	< 0.001		3.6 (1.0, 6.3)	0.02	0.052
*HW* 12	31	36.1 (28.4, 44.2)	48.53	< 0.001		3.1 (−0.7, 7.0)	14.27	0.100
CMA	16	28.5 (20.4, 37.2)	69.89	< 0.001		0.9 (−1.9, 3.7)	9.85	0.381

Note: There were no heatwave days identified in some of the 33 cities when adopting the heatwave definition of HW12 and CMA, so the number of cities was less than 33. *P* refers to the p-values from the Cochran’s Q test in the meta-regression models.

**Table 4:** Subgroup comparison of the effect of heat on mortality risk when adopted *HW*8 in 33 major cities in China from 2007 to 2013

Groups	Comparable group	Main effect				Added effect		
		Percentage change (95% *CI*)	Z	*P*		Percentage change (95% *CI*)	Z	*P*
Sex								
Male	vs. Female	23.3 (18.0, 28.8)	−2.92	0.004		2.2 (−0.5, 5.1)	−1.15	0.250
Female		41.4 (30.4, 53.4)				4.8 (1.5, 8.2)		
Age (Years)								
< 65	vs. 65−74	22.4 (13.4, 32.2)	−0.60	0.549		3.4 (0.3, 6.6)	0.79	0.430
65−74	vs. ⩾ 75	26.1 (19.0, 33.6)	−2.28	0.023		1.2 (−3.2, 5.8)	−0.83	0.407
⩾ 75	vs. < 65	41.1 (30.5, 52.5)	−2.55	0.011		3.7 (−0.1, 7.6)	−0.11	0.912
Educational attainments								
Illiterate	vs. Primary school	111.1(75.7, 153.5)	4.82	<0.001		7.4 (0.7, 14.5)	0.85	0.395
Primary school	vs. Secondary or higher	31.3 (23.5, 39.5)	3.03	0.002		4.0 (0.3, 7.9)	1.96	0.050
Secondary or higher	vs. Illiterate	16.1 (10.4, 22.2)	6.16	<0.001		−1.3 (−5.0, 2.5)	2.23	0.026

**Table 5:** Pooled main and added effects for mortality risk between coastal and inland cities with different heatwave definitions

*HW*	Main effect Percentage change *(95%CI)*			Added effect Percentage change (95%*CI*)	
	Coastal	Inland		Coastal	Inland
*HW*1	25.5 (15.9, 36.0)	21.3 (16.1, 26.6)		−0.5 (−3.4, 2.5)	0.6 (−1.5, 2.6)
*HW*2	29.8 (19.0, 41.6)	21.6 (15.9, 27.4)		−0.8 (−3.3, 1.8)	0.9 (−0.9, 2.7)
*HW*3	28.4 (19.3, 38.2)	22.5 (16.7, 28.7)		−0.3 (−5.7, 5.5)	0.7 (−1.5, 2.9)
*HW*4	26.6 (16.6, 37.4)	21.1 (15.5, 27.0)		1.2 (−1.4, 3.8)	1.2 (−0.3, 2.8)
*HW*5	29.3 (19.3, 40.2)	22.4 (16.4, 28.6)		0.2 (−2.8, 3.2)	2.0 (0.2, 3.8)
*HW*6	30.3 (20.2, 41.2)	22.2 (16.6, 28.0)		−1.5 (−7.5, 4.9)	3.3 (0.6, 6.0)
*HW*7	28.6 (16.5, 42.0)	24.5 (18.5, 30.7)		0.5 (−2.5, 3.7)	3.4 (1.3, 5.6)
*HW*8	32.8 (19.5, 47.5)	26.4 (19.5, 33.7)		−0.6 (−5.0, 3.9)	3.9 (0.9, 7.1)
*HW*9	33.6 (21.3, 47.3)	30.4 (23.3, 37.9)		−3.6 (−11.8, 5.4)	2.6 (−1.6, 6.9)
*HW*10	33.8 (19.3, 50.1)	31.8 (24.2, 40.0)		1.1 (−2.9, 5.2)	4.9 (2.4, 7.5)
*HW*11	37.4 (20.9, 56.1)	37.1 (27.9, 46.8)		−3.8 (−11.9, 5.1)	4.3 (1.2, 7.5)
*HW*12	34.9 (20.4, 51.1)	36.8 (27.4, 46.8)		3.0 (−5.3, 12.1)	2.7 (−1.5, 7.1)
CMA	20.7 (16.1, 25.5)	33.7 (21.6, 47.0)		1.1 (−6.2, 8.9)	0 (−2.9, 3.0)

## Highlights

- The main and added effect from heat co-existed in China.
- Both of the main and added effect could increase the mortality risk of population.
- Females, the elderly, the less educated and inland residents were more vulnerable.

## References

[R1] Aboubakri O, Khanjani N, Jahani Y, Bakhtiari B (2019). Attributable risk of mortality associated with heat and heat waves: a time-series study in Kerman, Iran during 2005–2017. Journal of Thermal Biology.

[R2] Anderson GB, Bell ML (2011). Heat waves in the United States: mortality risk during heat waves and effect modification by heat wave characteristics in 43 U.S. communities. Environmental Health Perspectives.

[R3] Arbuthnott KG, Hajat S (2017). The health effects of hotter summers and heat waves in the population of the United Kingdom: a review of the evidence. Environmental Health.

[R4] Barnett AG, Hajat S, Gasparrini A, Rocklöv J (2012). Cold and heat waves in the United States. Environmental Research.

[R5] Byers E, Gidden M, Leclère D, Balkovic J, Burek P, Ebi K, Greve P, Grey D, Havlik P, Hillers A (2018). Global exposure and vulnerability to multi-sector development and climate change hotspots. Environmental Research Letters.

[R6] Cai W, Zhang C, Zhang S, Ai S, Bai Y, Bao J, Chen B, Chang N, Chen H, Cheng L (2021). The 2021 China report of the Lancet Countdown on health and climate change: seizing the window of opportunity. Lancet Public Health.

[R7] Campbell S, Remenyi TA, White CJ, Johnston FH (2018). Heatwave and health impact research: a global review. Health & Place.

[R8] Center NMDI (2006). Coastal Administrative Areas Classification and Codes.

[R9] Chen K, Bi J, Chen J, Chen X, Huang L, Zhou L (2015). Influence of heat wave definitions to the added effect of heat waves on daily mortality in Nanjing, China. Science of the Total Environment.

[R10] Cheng J, Xu Z, Bambrick H, Su H, Tong S, Hu W (2018). Heatwave and elderly mortality: an evaluation of death burden and health costs considering short-term mortality displacement. Environment International.

[R11] Clogg CC, Petkova E, Haritou A (1995). Statistical methods for comparing regression coefficients between models. American Journal of Sociology.

[R12] Díaz J, Jordán A, García R, López C, Alberdi JC, Hernández E, Otero A (2002). Heat waves in Madrid 1986–1997: effects on the health of the elderly. International Archives of Occupational and Environmental Health.

[R13] Dimitrova A, Ingole V, Basagaña X, Ranzani O, Milà C, Ballester J, Tonne C (2021). Association between ambient temperature and heat waves with mortality in South Asia: systematic review and meta-analysis. Environment International.

[R14] Dong W, Zeng Q, Ma Y, Li G, Pan X (2016). Impact of heat wave definitions on the added effect of heat waves on cardiovascular mortality in Beijing, China. International Journal of Environmental Research and Public Health.

[R15] Druyan A, Makranz C, Moran D, Yanovich R, Epstein Y, Heled Y (2012). Heat tolerance in women: reconsidering the criteria. Aviation, Space, and Environmental Medicine.

[R16] Ebi KL, Capon A, Berry P, Broderick C, De Dear R, Havenith G, Honda Y, Kovats RS, Ma W, Malik A, Morris NB (2021). Hot weather and heat extremes: health risks. Lancet.

[R17] Gasparrini A (2011). Distributed lag linear and non-linear models in R: the package dlnm. Journal of Statistical Software.

[R18] Gasparrini A, Armstrong B (2011). The impact of heat waves on mortality. Epidemiology (Cambridge, Mass).

[R19] Gasparrini A, Armstrong B, Kenward MG (2012). Multivariate meta-analysis for non-linear and other multi-parameter associations. Statistics in Medicine.

[R20] Gasparrini A, Guo Y, Hashizume M, Lavigne E, Zanobetti A, Schwartz J, Tobias A, Tong S, Rocklöv J, Forsberg B (2015). Mortality risk attributable to high and low ambient temperature: a multicountry observational study. Lancet.

[R21] Gronlund CJ, Zanobetti A, Schwartz JD, Wellenius GA, O’neill MS (2014). Heat, heat waves, and hospital admissions among the elderly in the United States, 1992–2006. Environmental Health Perspectives.

[R22] Guo Y, Barnett AG, Pan X, Yu W, Tong S (2011). The impact of temperature on mortality in Tianjin, China: a case-crossover design with a distributed lag nonlinear model. Environmental Health Perspectives.

[R23] Guo Y, Gasparrini A, Armstrong B, Li S, Tawatsupa B, Tobias A, Lavigne E, De Sousa Zanotti Stagliorio Coelho M, Leone M, Pan X (2014). Global variation in the effects of ambient temperature on mortality: a systematic evaluation. Epidemiology (Cambridge, Mass).

[R24] Guo Y, Gasparrini A, Armstrong BG, Tawatsupa B, Tobias A, Lavigne E, Coelho M, Pan X, Kim H, Hashizume M (2017). Heat wave and mortality: a multicountry, multicommunity study. Environmental Health Perspectives.

[R25] Hajat S, Armstrong B, Baccini M, Biggeri A, Bisanti L, Russo A, Paldy A, Menne B, Kosatsky T (2006). Impact of high temperatures on mortality: is there an added heat wave effect?. Epidemiology (Cambridge, Mass).

[R26] Hajat S, Kosatky T (2010). Heat-related mortality: a review and exploration of heterogeneity. Journal of Epidemiology and Community Health.

[R27] Heo S, Bell ML, Lee JT (2019). Comparison of health risks by heat wave definition: applicability of wet-bulb globe temperature for heat wave criteria. Environmental Research.

[R28] Hersbach H, Bell B, Berrisford P, Hirahara S, Horányi A, Muñoz-Sabater J, Nicolas J, Peubey C, Radu R, Schepers D (2020). The ERA5 global reanalysis. Quarterly Journal of the Royal Meteorological Society.

[R29] Hoegh-Guldberg O, Jacob D, Taylor M, Bindi M, Brown S, Camilloni I, Diedhiou A, Djalante R, Ebi KL, Engelbrecht F (2018). Global Warming of 15°C: an IPCC Special Report on the Impacts of Global Warming of 15°C above Pre-industrial Levels and Related Global Greenhouse Gas Emission Pathways, in the Context of Strengthening the Global Esponse to the Threat of Climate Change, Sustainable Development, and Efforts to Eradicate Poverty.

[R30] Hu J, Wen Y, Duan Y, Yan S, Liao Y, Pan H, Zhu J, Yin P, Cheng J, Jiang H (2020). The impact of extreme heat and heat waves on emergency ambulance dispatches due to external cause in Shenzhen, China. Environmental Pollution.

[R31] Hu K, Guo Y, Hochrainer-Stigler S, Liu W, See L, Yang X, Zhong J, Fei F, Chen F, Zhang Y (2019). Evidence for urban-rural disparity in temperature-mortality relationships in Zhejiang Province, China. Environmental Health Perspectives.

[R32] Huang Y, Zhang T, Lou J, Wang P, Huang L (2022). Effective interventions on health effects of Chinese rural elderly under heat exposure. Frontiers of Environmental Science and Engeering.

[R33] Jay O, Capon A, Berry P, Broderick C, De Dear R, Havenith G, Honda Y, Kovats RS, Ma W, Malik A (2021). Reducing the health effects of hot weather and heat extremes: from personal cooling strategies to green cities. Lancet.

[R34] Kim EJ, Kim H (2017). Effect modification of individual- and regional-scale characteristics on heat wave-related mortality rates between 2009 and 2012 in Seoul, South Korea. Science of the Total Environment.

[R35] Lange S, Volkholz J, Geiger T, Zhao F, Vega I, Veldkamp T, Reyer CPO, Warszawski L, Huber V, Jägermeyr J (2020). Projecting exposure to extreme climate impact events across six event categories and three spatial scales. Earth’s Future.

[R36] Lee W, Choi HM, Lee JY, Kim DH, Honda Y, Kim H (2018). Temporal changes in mortality impacts of heat wave and cold spell in Korea and Japan. Environment International.

[R37] Lee WK, Lee HA, Lim YH, Park H (2016). Added effect of heat wave on mortality in Seoul, Korea. International Journal of Biometeorology.

[R38] Nori-Sarma A, Benmarhnia T, Rajiva A, Azhar GS, Gupta P, Pednekar MS, Bell ML (2019). Advancing our understanding of heat wave criteria and associated health impacts to improve heat wave alerts in developing country settings. International Journal of Environmental Research and Public Health.

[R39] Paternoster R, Brame R, Mazerolle P, Piquero A (1998). Using the correct statistical test for the equality of regression coefficients. Criminology.

[R40] Qiao Z, Guo Y, Yu W, Tong S (2015). Assessment of short- and long-term mortality displacement in heat-related deaths in Brisbane, Australia, 1996–2004. Environmental Health Perspectives.

[R41] Romanello M, Mcgushin A, Di Napoli C, Drummond P, Hughes N, Jamart L, Kennard H, Lampard P, Solano Rodriguez B, Arnell N (2021). The 2021 report of the Lancet Countdown on health and climate change: code red for a healthy future. Lancet.

[R42] Sera F, Armstrong B, Tobias A, Vicedo-Cabrera AM, Åström C, Bell ML, Chen BY, de Sousa Zanotti Stagliorio Coelho M, Matus Correa P, Cruz JC (2019). How urban characteristics affect vulnerability to heat and cold: a multi-country analysis. International Journal of Epidemiology.

[R43] Sherbakov T, Malig B, Guirguis K, Gershunov A, Basu R (2018). Ambient temperature and added heat wave effects on hospitalizations in California from 1999 to 2009. Environmental Research.

[R44] Son JY, Lee JT, Anderson GB, Bell ML (2012). The impact of heat waves on mortality in seven major cities in Korea. Environmental Health Perspectives.

[R45] Son JY, Liu JC, Bell ML (2019). Temperature-related mortality: a systematic review and investigation of effect modifiers. Environmental Research Letters.

[R46] Sun Z, Wang Q, Chen C, Yang Y, Yan M, Du H, Chen K, Ji JS, Li T (2021). Projection of temperature-related excess mortality by integrating population adaptability under changing climate, China, 2050s and 2080s. China CDC Weekly.

[R47] Tan ZH (2011). The Calculation and Simulation of Chinese North-South Demarcation Based on GIS.

[R48] Tong S, Fitzgerald G, Wang XY, Aitken P, Tippett V, Chen D, Wang X, Guo Y (2015). Exploration of the health risk-based definition for heatwave: a multi-city study. Environmental Research.

[R49] Vicedo-Cabrera AM, Scovronick N, Sera F, Roye D, Schneider R, Tobias A, Astrom C, Guo Y, Honda Y, Hondula DM (2021). The burden of heat-related mortality attributable to recent human-induced climate change. Nature Climate Change.

[R50] Viechtbauer W (2010). Conducting Meta-Analyses in R with the metafor Package. Journal of Statistical Software.

[R51] Wang JF, Hu Y (2012). Environmental health risk detection with GeogDetector. Environmental Modelling & Software.

[R52] Wang JF, Li XH, Christakos G, Liao YL, Zhang T, Gu X, Zheng XY (2010). Geographical detectors-based health risk assessment and its application in the neural tube defects study of the Heshun Region, China. International Journal of Geographical Information Science.

[R53] Wang JF, Zhang TL, Fu BJ (2016). A measure of spatial stratified heterogeneity. Ecological Indicators.

[R54] Wickham H (2016). ggplot2–Elegant Graphics for Data Analysis.

[R55] Xu Z, Fitzgerald G, Guo Y, Jalaludin B, Tong S (2016). Impact of heatwave on mortality under different heatwave definitions: a systematic review and meta-analysis. Environment International.

[R56] Xue T, Zheng Y, Tong D, Zheng B, Li X, Zhu T, Zhang Q (2019). Spatiotemporal continuous estimates of PM_2.5_ concentrations in China, 2000–2016: a machine learning method with inputs from satellites, chemical transport model, and ground observations. Environment International.

[R57] Yang J, Yin P, Sun J, Wang B, Zhou M, Li M, Tong S, Meng B, Guo Y, Liu Q (2019). Heatwave and mortality in 31 major Chinese cities: definition, vulnerability and implications. Science of the Total Environment.

[R58] Yang J, Zhou M, Ren Z, Li M, Wang B, Liu L, Ou CQ, Yin P, Sun J, Tong S, Wang H (2021). Projecting heat-related excess mortality under climate change scenarios in China. Nature Communications.

[R59] Yin P, Chen R, Wang L, Liu C, Niu Y, Wang W, Jiang Y, Liu Y, Liu J, Qi J, You J (2018). The added effects of heatwaves on cause-specific mortality: a nationwide analysis in 272 Chinese cities. Environment International.

[R60] Zhao Q, Guo Y, Ye T, Gasparrini A, Tong S, Overcenco A, Urban A, Schneider A, Entezari A, Vicedo-Cabrera AM (2021). Global, regional, and national burden of mortality associated with non-optimal ambient temperatures from 2000 to 2019: a three-stage modelling study. Lancet Planetary Health.

