# Supplementary material for: The main and added effects of heat on mortality in 33 Chinese cities from 2007 to 2013
Source: Front Environ Sci Eng. Author manuscript; Available in PMC 2024 Oct 24. (PMC7616734; doi:10.1007/s11783-023-1681-5)
Supplement: Supplementary Material [file EMS199299-supplement-Supplementary_Material.pdf]

Supplementary Material

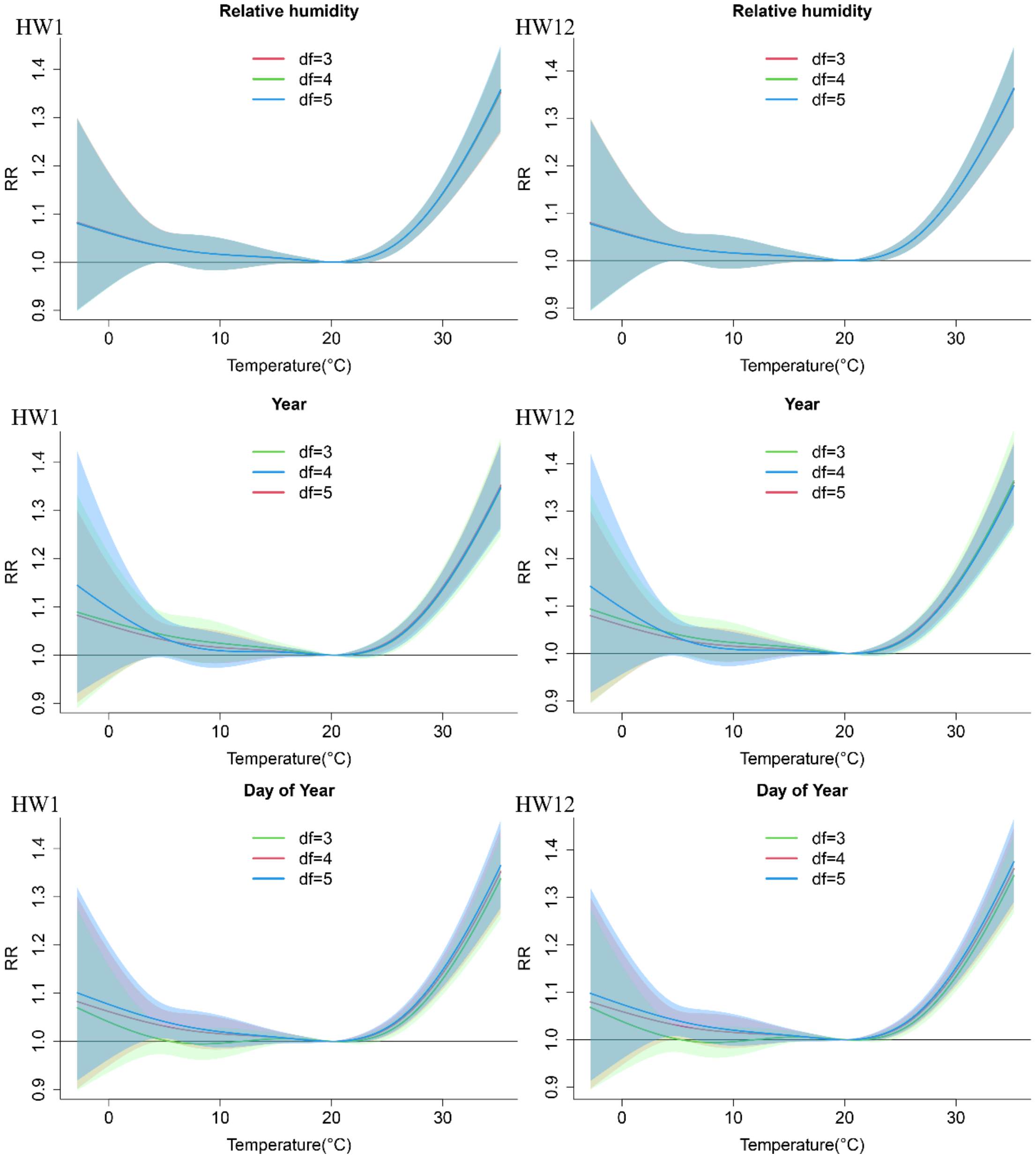

Note: There was not much difference in sensitivity analysis when altering the degrees of freedom (df = 3–5) for relative humidity, where the lines and areas in figures were almost overlap.

Figure S1. Sensitivity analysis when altering the degrees of freedom (df = 3–5) for relative humidity, year and day of year in the model

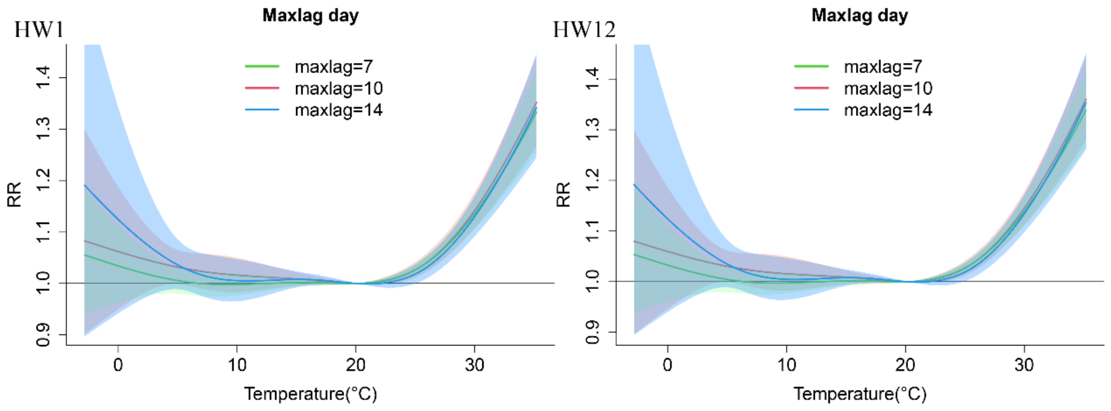

Figure S2. Sensitivity analysis when changing the maximum lag day into 7 and 14 in the model

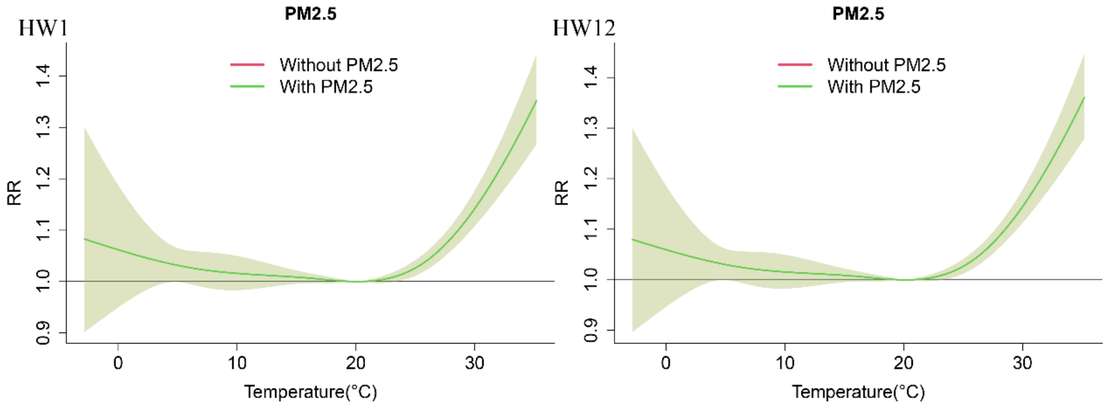

Note: There was not much difference in sensitivity analysis between models with air pollutants and without air pollutants, where the lines and areas in figures were almost overlap.

Figure S3. Sensitivity analysis between models with air pollutants and without air pollutants

Table S1. The distribution of 33 cities in divisions for the south and the north, and the coastal cities and the inland cities

Divisions		No. Cities	Names of Cities
The south and the north	The north	15	Beijing, Taiyuan, Tianjin, Yinchuan, Zhengzhou, Xining, Jinan, Lanzhou, Xi’an, Changchun, Shenyang, Shijiazhuang, Harbin, Urumqi and Hohhot
	The south	17	Hangzhou, Shenzhen, Guangzhou, Hefei, Chengdu, Wuhan, Changsha, Guiyang, Fuzhou, Nanjing, Ningbo, Kunming, Shanghai, Nanchang, Chongqing, Haikou and Nanning
The coastal cities and the inland cities	The coastal cities	8	Hangzhou, Shenzhen, Tianjin, Fuzhou, Ningbo, Shanghai, Guangzhou and Haikou
	The inland cities	25	Beijing, Taiyuan, Hefei, Chengdu, Wuhan, Changsha, Guiyang, Yinchuan, Zhengzhou, Lhasa, Nanjing, Xining, Jinan, Kunming, Lanzhou, Xi’an, Nanchang, Chongqing, Shenyang, Changchun, Nanning, Shijiazhuang, Urumqi, Hohhot and Harbin

Note: Because Lhasa is located on the Qinghai-Tibet Plateau, it is neither belong to the south nor the north, and it is not included in Spatial stratified heterogeneity analysis between the north and the south.

Table S2. The statistics of average daily death number, daily mean temperature, relative humidity and $PM_{2.5}$ concentration in 33 Chinese cities in warm season from 2007 to 2013

Cities	Average daily death number	daily mean temperature (°C)				RH (%)	$PM_{2.5}$ (μg/m^3)
		Mean	P25	Median	P75		
Beijing	180	22.44	20.17	22.97	25.14	63.73	68.93
Tianjin	149	24.31	22.17	24.91	26.93	65.31	72.90
Shijiazhuang	53	23.37	21.11	23.72	26.23	66.27	83.14
Taiyuan	25	19.08	16.69	19.41	22.02	62.38	54.02
Hohhot	14	18.25	15.50	18.95	21.37	51.01	34.22
Shenyang	100	21.58	18.91	22.24	24.61	68.44	52.97
Changchun	53	20.05	16.75	20.82	23.66	66.78	37.37
Harbin	145	18.64	15.31	19.43	22.21	71.46	30.41
Shanghai	147	25.80	22.97	26.15	29.07	77.51	49.27
Nanjing	85	25.56	22.92	25.92	28.51	75.95	56.80
Hangzhou	21	25.71	22.86	26.12	28.61	73.75	43.73
Ningbo	12	25.71	22.90	26.23	28.78	78.64	36.32
Hefei	48	25.57	22.85	25.85	28.35	75.36	55.43
Fuzhou	14	25.45	23.89	26.06	27.50	80.58	31.96
Nanchang	17	27.54	24.89	27.96	30.46	74.99	47.57
Jinan	58	23.97	21.56	24.46	26.74	69.92	71.20
Zhengzhou	36	24.39	21.67	24.92	27.25	68.31	73.28
Wuhan	26	26.32	23.87	26.71	29.14	75.86	60.14
Changsha	75	26.51	23.74	26.92	29.67	73.64	54.22
Guangzhou	74	27.68	26.42	27.86	29.22	79.40	48.05
Shenzhen	10	27.68	26.70	27.89	28.93	82.11	35.73
Nanning	43	27.40	26.31	27.73	28.83	79.93	35.73
Haikou	4	27.16	26.39	27.22	27.96	83.11	27.35
Chongqing	239	23.68	21.12	23.95	26.41	76.21	55.12
Chengdu	158	22.49	20.51	22.79	24.80	75.53	54.06
Guiyang	9	21.74	19.62	22.37	24.18	77.83	38.49
Kunming	69	19.56	18.51	19.83	20.98	74.33	27.03
Lhasa	2	7.15	5.58	7.77	9.15	67.22	34.55
Xi’an	44	20.88	18.36	21.39	23.82	72.18	49.27
Lanzhou	9	17.29	14.66	17.65	20.19	56.58	37.91
Xining	14	10.36	8.20	10.57	12.64	68.77	36.21
Yinchuan	14	20.40	17.67	20.79	23.66	49.12	37.74
Urumqi	14	14.17	11.47	14.72	17.16	51.73	32.79

Note: P25 and P75 refer to 25th percentile and 75th percentile, respectively. RH and $PM_{2.5}$ refer to daily mean relative humidity and daily average concentration of $PM_{2.5}$, respectively.

Table S3. Pooled main and added effects for mortality risk with different heatwave definitions in subgroups

HW	Gender		Age (Years)			Educational attainments		
	Male	Female	<65	65-74	≥75	Illiterate	Primary school	Secondary or higher
**Main Effect**								
HW1	19.8 (15.3, 24.6)	32.0 (24.3, 40.2)	17.9 (11.8, 24.4)	20.4 (13.3, 27.9)	33.2 (25.9, 41.0)	87.3 (60.6, 118.5)	27.1 (20.3, 34.4)	15.0 (9.0, 21.4)
HW2	20.7 (15.8, 25.8)	32.4 (24.4, 40.9)	18.0 (11.6, 24.7)	22.6 (16.3, 29.3)	34.9 (26.7, 43.6)	88.7 (61.7, 120.2)	27.4 (20.3, 35.0)	13.1 (8.3, 18.0)
HW3	20.9 (16.3, 25.7)	31.9 (24.0, 40.3)	16.9 (10.2, 24.1)	22.6 (15.1, 30.6)	36.0 (27.7, 44.9)	84.8 (60.3, 113.0)	27.6 (20.9, 34.6)	14.2 (9.5, 19.0)
HW4	20.5 (16.4, 24.8)	32.8 (24.4, 41.7)	17.1 (10.9, 23.6)	22.7 (15.7, 30.2)	34.6 (26.0, 43.8)	93.9 (64.6, 128.5)	27.8 (20.2, 35.9)	13.5 (9.3, 17.9)
HW5	22.0 (16.9, 27.3)	33.4 (25.0, 42.4)	18.8 (11.5, 26.6)	25.7 (19.0, 32.8)	36.1 (27.5, 45.4)	93.2 (65.0, 126.1)	27.0 (20.9, 33.4)	13.3 (9.3, 17.4)
HW6	21.8 (16.9, 27.0)	35.1 (26.2, 44.6)	17.8 (9.9, 26.3)	22.2 (15.4, 29.4)	37.1 (28.2, 46.5)	89.2 (63.6, 118.8)	28.0 (20.5, 35.9)	15.8 (10.9, 20.8)
HW7	21.7 (17.2, 26.3)	36.2 (26.4, 46.8)	20.8 (12.9, 29.3)	23.6 (17.3, 30.1)	38.7 (29.0, 49.2)	103.8 (70.1, 144.3)	28.3 (21.3, 35.8)	15.2 (10.2, 20.4)
HW8	23.3 (18.0, 28.8)	41.4 (30.4, 53.4)	22.4 (13.4, 32.2)	26.1 (19.0, 33.6)	41.1 (30.5, 52.5)	111.1 (75.7, 153.5)	31.3 (23.5, 39.5)	16.1 (10.4, 22.2)
HW9	24.8 (19.7, 30.1)	46.9 (34.8, 60.0)	24.9 (14.5, 36.3)	28.2 (19.8, 37.1)	46.2 (35.2, 57.9)	120.8 (85.5, 162.9)	33.1 (24.7, 42.2)	20.1 (13.1, 27.5)
HW10	25.6 (19.9, 31.6)	46.0 (34.9, 58.1)	25.6 (15.1, 37.0)	26.6 (18.1, 35.7)	48.2 (36.5, 60.9)	127.3 (87.2, 176.2)	34.1 (24.8, 44.1)	20.1 (12.9, 27.7)
HW11	29.5 (22.5, 37.0)	53.1 (39.3, 68.2)	31.5 (18.8, 45.5)	30.8 (20.8, 41.6)	54.0 (40.6, 68.8)	137.0 (95.5, 187.2)	39.2 (27.7, 51.7)	23.6 (15.7, 32.2)
HW12	28.6 (22.2, 35.3)	51.8 (37.6, 67.4)	32.7 (19.4, 47.4)	32.2 (21.2, 44.1)	47.6 (36.4, 59.9)	152.8 (103.3, 214.5)	37.5 (26.7, 49.1)	18.9 (11.4, 27.1)
CMA	23.9 (18.2, 30.0)	32.2 (25.8, 39.0)	19.2 (9.3, 30.0)	27.7 (19.0, 37.0)	38.5 (28.2, 49.5)	99.8 (52.9, 161.0)	30.5 (21.2, 40.5)	14.1 (6.0, 22.8)
**Added Effect**								
HW1	0.8 (-1.1, 2.7)	-0.1 (-1.8, 1.5)	-0.4 (-2.6, 1.8)	1.0 (-1.3, 3.4)	0.6 (-1.2, 2.4)	1.6 (-3.1, 6.4)	2.0 (-0.3, 4.3)	-0.7 (-2.5, 1.2)
HW2	0.3 (-1.3, 1.9)	0.8 (-1.4, 3.0)	-0.4 (-2.8, 2.0)	1.7 (-1.4, 4.9)	0.5 (-1.8, 3.0)	2.1 (-2.6, 6.9)	0.9 (-1.8, 3.8)	-0.4 (-2.6, 1.7)
HW3	0.3 (-1.6, 2.1)	1.6 (-0.4, 3.7)	-0.1 (-2.8, 2.7)	2.6 (-0.7, 6.1)	0.8 (-1.2, 2.9)	3.9 (-1.6, 9.7)	0.9 (-1.7, 3.7)	-1.8 (-4.7, 1.3)
HW4	1.4 (-0.2, 3.1)	1.0 (-1.0, 2.9)	2.0 (-0.2, 4.2)	0.5 (-2.1, 3.1)	1.2 (-0.5, 3.0)	1.7 (-1.6, 5.1)	2.3 (-0.1, 4.8)	0.7 (-1.4, 2.8)
HW5	1.1 (-0.8, 3.0)	2.3 (0.1, 4.4)	1.5 (-1.0, 4.1)	0.3 (-3.4, 4.2)	1.9 (-0.1, 4.0)	3.4 (-0.8, 7.9)	0.6 (-2.4, 3.7)	0.9 (-1.6, 3.5)
HW6	2.0 (-0.1, 4.3)	3.8 (0.9, 6.7)	3.7 (0.5, 7.0)	2.2 (-1.6, 6.2)	2.6 (-0.1, 5.5)	5.5 (-1.2, 12.6)	2.9 (-0.2, 6.1)	-0.9 (-4.6, 2.9)
HW7	2.1 (-0.4, 4.7)	3.9 (1.6, 6.3)	1.8 (-1.3, 5.1)	0 (-3.6, 3.7)	4.3 (1.7, 7.0)	5.5 (0.6, 10.7)	3.4 (0.2, 6.8)	0.3 (-2.7, 3.3)

HW8	2.2 (-0.5, 5.1)	4.8 (1.5, 8.2)	3.4 (0.3, 6.6)	1.2 (-3.2, 5.7)	3.7 (-0.1, 7.6)	7.4 (0.7, 14.5)	4.0 (0.3, 7.9)	-1.3 (-5.0, 2.5)
HW9	2.0 (-1.9, 5.9)	3.3 (-0.8, 7.6)	3.1 (-1.8, 8.2)	0.6 (-4.5, 5.9)	3.0 (-1.5, 7.8)	4.6 (-4.3, 14.3)	6.5 (2.5, 10.7)	-5.7 (-10.8, -0.3)
HW10	3.1 (0.3, 5.9)	5.1 (1.2, 9.2)	3.5 (0.1, 7.0)	3.7 (-0.3, 7.9)	3.5 (-0.4, 7.5)	11.7 (4.2, 19.8)	6.5 (2.9, 10.2)	-5.0 (-8.0, -1.9)
HW11	2.6 (-0.7, 6.0)	4.9 (1.4, 8.6)	3.6 (-1.0, 8.5)	2.3 (-2.9, 7.8)	2.4 (-1.9, 7.0)	8.3 (-1.3, 18.9)	7.6 (3.1, 12.3)	-7.4 (-12.1, -2.5)
HW12	2.9 (-1.8, 7.7)	4.8 (0.4, 9.4)	1.3 (-6.1, 9.3)	0.3 (-5.9, 6.9)	7.3 (3.0, 11.6)	7.9 (-1.4, 18.0)	9.2 (3.4, 15.3)	-7.4 (-13.3, -1.1)
CMA	0.5 (-3.5, 4.6)	1.9 (-1.6, 5.5)	1.2 (-4.1, 6.8)	-2.0 (-7.6, 3.9)	2.3 (-1.0, 5.6)	-1.2 (-9.7, 8.0)	-0.4 (-4.9, 4.2)	4.7 (-4.0, 14.2)

Note: HW refers to the heat wave definition.

Table S4. Spatial heterogeneity of heat effect on mortality risk in different divisions with different heatwave definitions in China from 2007-2013

HW	Main effect				Added effect			
	South and north		Coastal and inland		South and north		Coastal and inland	
	q	*p*	*q*	*p*	*q*	*p*	*q*	*p*
HW1	0.0460	0.2541	0.0022	0.8114	0.0057	0.6836	0.0363	0.3097
HW2	0.0498	0.2364	0.0020	0.8193	0.0050	0.7008	0.0343	0.3230
HW3	0.0546	0.2159	0.0014	0.8508	0.0335	0.3321	0.0004	0.9069
HW4	0.0429	0.2700	0.0015	0.8426	0.0002	0.9325	0.0108	0.5667
HW5	0.0469	0.2498	0.0031	0.7790	< 0.0001	0.9945	0.0098	0.5892
HW6	0.0497	0.2367	0.0020	0.8194	0.0586	0.2067	0.0359	0.3160
HW7	0.0399	0.2863	0.0017	0.8326	0.0685	0.1729	0.0045	0.7118
HW8	0.0484	0.2424	0.0039	0.7569	0.0009	0.8720	0.0460	0.2637
HW9	0.0351	0.3159	0.0009	0.8796	0.0015	0.8329	0.0251	0.4085
HW10	0.0393	0.2895	0.0040	0.7564	< 0.0001	0.9702	0.1469	0.0606
HW11	0.0314	0.3417	0.0022	0.8181	0.0094	0.6003	0.1242	0.1002
HW12	0.0062	0.6800	0.0319	0.3396	0.0731	0.1781	0.0085	0.6211
CMA	0.1001	0.2485	0.1285	0.1876	0.0368	0.4918	0.1200	0.2508

Note: HW refers to the heat wave definition.

Table S5. Pooled main and added effects for mortality risk between north and south cities with different heatwave definitions

HW	Main effect		Added effect	
	North	South	North	South
HW1	22.2 (18.9, 25.6)	26.8 (16.4, 38.0)	0.1 (-2.8, 3.0)	0 (-1.8, 1.8)
HW2	21.6 (16.8, 26.6)	30.7 (18.8, 43.8)	0.8 (-1.7, 3.4)	-0.1 (-2.0, 1.8)
HW3	21.8 (17.5, 26.3)	31.6 (19.6, 44.7)	1.5 (-1.3, 4.3)	-0.1 (-2.8, 2.7)
HW4	21.9 (16.2, 27.9)	25.8 (15.9, 36.6)	1.2 (-0.7, 3.1)	1.3 (-0.7, 3.2)
HW5	22.7 (17.1, 28.6)	30.0 (18.7, 42.3)	1.6 (-0.7, 3.9)	1.6 (-0.6, 3.7)
HW6	22.7 (17.5, 28.1)	31.1 (19.8, 43.6)	4.7 (1.8, 7.6)	0.6 (-2.9, 4.2)
HW7	26.0 (21.2, 30.9)	29.7 (17.8, 42.7)	2.2 (-0.2, 4.7)	3.3 (0.6, 6.1)
HW8	26.8 (21.2, 32.6)	35.3 (21.6, 50.7)	2.5 (-1.5, 6.8)	2.7 (-1.0, 6.5)
HW9	32.5 (27.9, 37.3)	35.2 (22.6, 49.2)	1.5 (-5.0, 8.3)	0.5 (-4.4, 5.6)
HW10	33.5 (28.3, 38.9)	38.3 (22.8, 55.8)	3.2 (0, 6.5)	4.4 (1.3, 7.5)
HW11	37.9 (29.8, 46.6)	40.5 (25.1, 57.7)	3.2 (-1.3, 7.8)	3.5 (0.2, 7.0)
HW12	37.2 (27.7, 47.4)	37.1 (24.0, 51.6)	-0.1 (-7.1, 7.5)	4.2 (-0.4, 9.1)
CMA	26.8 (14.0, 41.1)	28.7 (20.5, 37.4)	1.8 (-4.2, 8.1)	0.7 (-2.7, 4.1)

Note: HW refers to the heat wave definition.

Table S6. Sensitivity analysis for the added effect

Variables	Values	Percentage change in RR (95%CI)	
		HW1	HW12
Original model	0	0.2 (-1.4 to 1.9)	3.1 (-0.7 to 7)
Year.df	3	0.5 (-1.3 to 2.4)	2.7 (-1.2 to 6.7)
	4	0.4 (-1.3 to 2.1)	3.6 (-0.2 to 7.5)
Doy.df	3	0.3 (-1.3 to 1.9)	3.3 (-0.5 to 7.3)
	5	0.3 (-1.3 to 1.9)	3.1 (-0.7 to 7)
RH.df	4	0.3 (-1.3 to 1.9)	3.4 (-0.3 to 7.3)
	5	0.3 (-1.4 to 1.9)	3.6 (-0.1 to 7.4)
Maxlag	7	0.3 (-1.3 to 1.9)	3.2 (-0.6 to 7.1)
	14	0.3 (-1.4 to 2)	3.1 (-0.7 to 7.1)
Without $PM_{2.5}$		0.3 (-1.4 to 1.9)	3.1 (-0.7 to 7)

Note: Year.df, Doy.df and RH.df refer to the df for year, day of year, and relative humidity in the model, respectively. Maxlag refers to the maximum lag day of $Temp_{t,l}$.

Table S7. QAIC for the four temperature metrics in the exposure-response model with different heatwave definitions

HW	Tmean	Tmax	Tmin	AT
HW1	235731.2	236047.4	236454.6	236091.5
HW2	235757.4	236046.8	236450.5	236088.9
HW3	235750.2	236022.1	236429.7	236056.2
HW4	235800.8	236046.1	236431.1	236083.8
HW5	235749.4	236080.9	236431.0	236070.0
HW6	235721.4	236073.5	236427.7	236006.8
HW7	235682.3	236029.5	236435.1	236010.9
HW8	235644.6	236022.1	236396.5	235987.6
HW9	235659.9	236000.4	236284.6	235907.1
HW10	235750.3	236036.5	236424.2	236037.8
HW11	235716.2	235971.4	236358.7	235951.5
HW12	235729.8	235985.0	236371.9	236026.7
CMA	235841.6	236108.7	236523.2	236125.2
Sum	3064535.1	3068470.5	3073418.9	3068444.0

Note: The Akaike Information Criterion for quasi-Poisson (Q-AIC) was used to assess the goodness of model fits among 13 heatwave definitions and 4 temperature metrics. The sum of Q-AIC values for each temperature metric in all heatwave definitions from all models in 33 cities were compared. Then the optimal temperature metric used in this study was determined when the sum was minimum. Tmean, Tmax, Tmin and AT refer to daily mean temperature, daily maximum temperature, daily minimum temperature and apparent temperature, respectively. The AT was calculated by the common meteorological indicators, including daily mean temperature, relative humidity and barometric pressure using the following equations.

$$AT=T+0.33*e-0.70*WS-4.00 \quad (1)$$

$$e=RH/100*6.105*\exp(17.27*T/(237.7+T)) \quad (2)$$

In eq. (1), T is the daily mean temperature (°C), e water vapor pressure (hPa) and WS average wind velocity (m/s). The water vapor pressure e is calculated with the daily mean temperature and relative humidity using eq. (2); RH denotes relative humidity (%).
